# Walsh's Physician's Call Book

**Published:** 1881-01

**Authors:** 


### BIBLIOGRAPHICAL.

*Walsh's Physicians Call Book* has been used for some time by us as an *appointment book*, answering well for that purpose, and containing a mass of information valuable to all. The table of metric doses as proportioned to the ordinary system, is very useful. It is, in addition to being an excellent blank book, a perfect compendium of what most physicians and dentists would like to have at hand continually. H.

